# Multiple Receptor Subtypes for the CGRP Super-Family

**DOI:** 10.1100/tsw.2001.436

**Published:** 2001-11-21

**Authors:** R. Quirion, J.-G. Chabot Chabot, Y. Dumont

**Affiliations:** Douglas Hospital Research Center, McGill University, Montreal, QC, Canada H4H 1R3,

## Abstract

Molecular evidence for the existence of multiple receptors for CGRP has been rather difficult to obtain. Over 10 years after suggesting the existence of at least two classes (CGRP1 and CGRP2) of CGRP receptors on the basis of pharmacological data[1], molecular data on the CGRP2 receptor subtype are still lacking as well as potent and selective antagonists. The situation is somewhat different for the functional CGRP1 subtype which is likely composed of diverse subunits CRLR, RAMP1 and possibly RCP[2]. Moreover, BIBN 4096BS was recently reported as the first nonpeptide highly potent CGRP1 receptor antagonist[3]. However, in situ hybridization and receptor autoradiographic data have clearly shown the existence of major mismatches (e.g., cerebellum) between the discrete localization of CRLR, RAMP1, and specific CGRP binding sites supporting the existence of CGRP receptor subtypes. Functional studies have also provided evidence in that regard (for a recent review: [4]). Accordingly, additional studies aiming at cloning additional CGRP receptors are certainly warranted. Similarly, recent evidence from various laboratories including ours suggests the existence of more than one class (CRLR and RAMP2) of adrenomedullin receptors at least in the rat brain. In contrast, most evidence suggests the existence of a single class of amylin receptors. In brief, it appears that multiple receptors or receptor complexes do exist for CGRP and related peptides but their composition is apparently unique among the GPCR super-family and additional data are needed to fully establish the molecular organization of each subtype. Supported by CIHR of Canada.

